# Transportation, Environmental Degradation, and Health Dynamics in the United States and China: Evidence From Bootstrap ARDL With a Fourier Function

**DOI:** 10.3389/fpubh.2022.907390

**Published:** 2022-06-29

**Authors:** Meng-Chen Lin, Cheng-Feng Wu

**Affiliations:** ^1^School of Business Administration, Hubei University of Economics, Wuhan, China; ^2^School of Business, Wuchang University of Technology, Wuhan, China; ^3^Research Center of Hubei Logistics Development, Hubei University of Economics, Wuhan, China

**Keywords:** bootstrap ARDL approach, Fourier function approximation, transportation, environmental degradation, health, environmental Kuznets curve (EKC)

## Abstract

Transportation and environmental degradation, with indirect and direct effects, play a significant role in determining the health of a nation's citizens. This study uses bootstrap ARDL with a Fourier function to examine transportation, environmental degradation, and health dynamics in the United States and China. In the long run, the results support the cointegration relationship between transportation, environmental degradation, and health in both countries. The results show the contingency of the causality where a negative impact of transportation on environmental degradation exists in the United States while a positive impact exists in China. The effect of environmental degradation on health is negative in the United States while a positive effect exists in China. Regarding the causal direction between the variables of interest, the implications provide policymakers in developing strategy and policy for sustainable development.

## Introduction

Human health has become an *ad hoc* topic and development strategy for a country to improve the quality of human capital since previous studies emphasized that human capital accumulation related to production factors affects the growth of an economy (1, 2). To have better human health in a country, there has been a growing trend to assess the impact of environmental aspects on a regular basis (3, 4). In fact, transportation as an engine of economic growth directly affects the environment through the lens of the environmental Kuznets curve (EKC) (5–7). Therefore, relentlessly reviewing the dynamic relationship between transportation, environment, and health is very crucial to enhancing the growth of an economy.

With the expansion in economic development and international trade, global transport services have experienced a growth in delivery services, such as freight and passenger. Access to adequate transportation services in a supply chain is an essential determinant of progress and expansion for society and economics in a country (8). Transportation is viewed as one of the fundamentals for supporting the development of a nation since transportation shows its added value in a supply chain and is beneficial to economic growth. As a large component of the transportation sector, air transport not only allows quick mobility for freight service but also increases social connectedness for passenger service, and contributes to considerable outputs in economics (9). According to the International Air Transport Association, air cargo delivers about one-third of global trade value annually, making it a critical contribution to the supply chain solution (10). Nevertheless, within the globalization context, transport activities extend to the problem that a negative effect of the activities influences the environment in human society. The business activities related to freight transport as a major cause of air pollution generate vast CO2 emissions in the environment (11). The growth in the amount of CO2 emissions in the environment leads to harmful effects on human health (4, 12) and results in climate change (13).

The quality of the environment varies depending on the development of a country. The EKC theory has been highlighted as a helpful theoretical fundamental to identify the impact of environments (14, 15). In the framework of the EKC, an economy in the early stage of economic development leads to environmental degradation. Specifically, transportation activities as an engine of economics eject harmful gas into the environment. However, the development of the economy steps to the next stage where human beings with higher income request a better quality of life. Thus, humans in society and enterprises in economic activities pursue a better environment triggered by the decarbonization policy. Investigating the transport-induced EKC hypothesis, Erdogan et al. (16) affirmed that the connection between transportation and the deterioration in environmental quality since transportation is an important manner of the growth of an economy. The quality of the environment will deteriorate at the beginning where the industrial sector relying on transportation is the major contributor to the economic development; however, with the growth of an economy, the condition of the environment is improved since the wealth of the residents is improved and they focus on their environmental quality for a better quality of life.

According to a study by Mujtaba and Shahzad (17), the effect of the environment on health is discussed within the aspects of healthcare expenditure and health status. Polluted environments could decrease the immune system of humans and generate various diseases, such as respiratory diseases and lung cancer (18–20). Thus, humans in society pay the price for polluted environments through healthcare expenditure (19) and health status (20). Based on the foundation of the EKC theory, Moosa and Pham (21) and Moosa et al. (22) proposed a stylized representation regarding the association between healthcare expenditures and environmental degradation. They showed that income in an economy is an important connection when discussing the association between the deterioration in environmental quality and healthcare expenditure. Additionally, human beings would perform defensive behavior through activities where they aim to avoid, prevent, and mitigate the negative effect of the environment when facing the challenge of environmental pollution. Williams et al. (23) addressed the definition of health expenditures as follows: “health expenditures include the provision of health services (preventive and curative), family planning activities, nutrition activities, and emergency aid designated for health”. Human beings with abundant income demand better nutrition, medical care, and medicines since human beings in society have more consciousness of better quality of life along with the growth of the economy (24). Moreover, human beings with a better quality of life could increase preventive health expenditures to strengthen their health itself. Thus, the effect of the environment could positively or negatively alter health expenditures.

Previous studies have investigated the transport-induced EKC hypothesis (25–27) in a stream and the nexus between environment and health in another stream (3, 4). However, limited studies combine the transport-induced EKC with the nexus between environment and health to examine the health effect. Since firms provide time value in the competitive priorities to customers, air transportation is an important driver for the performance of a supply chain. Also, air freight contributes to a large part of trade value worldwide (10). Thus, this study collects air freight as the proxy variable of transportation. Within the field of social science including economics (28–33), environment (14, 34–37), and transportation (38–42) categories, the indicators perform the attributes of smooth and sharp shifts in the trend of time series. Smooth and sharp breaks are important and considered in previous studies since any effects of shocks on the indicators would affect the results of a model (43). Thus, without considering structural breaks in a model, the results may show bias and conclude with a misleading implication (44).

To the best of our knowledge, this is the first study that investigates the dynamics between transportation, environment, and health in the United States and China by using the bootstrap autoregressive-distributed lag test with a Fourier function. This study contributes to the existing literature in the following aspects. First, rather than simply measuring environmental indicators or health effects, this study concerns the transport-induced EKC and the environment-health nexus to examine how transportation and environmental degradation, with indirect and direct effects, play a significant role in determining health in a country. Second, the model employs a Fourier function that allows for capturing structural breaks. The effect of structural breaks is usually ignored in causality and unit root tests (43, 44). A Fourier function is used to consider multiple structural changes where the number, location, or forms (45). Third, two major countries that contribute to economic development and inject major CO2 emissions into the world are investigated in this study and this paper offers policy recommendations and motivates the government to be concerned about the development of a healthy and productive economy. The rest of the work is organized in the following manner: Section Data and Method includes data and method. Results are covered in Section Results. Discussion and conclusion are included in Section Discussion and Conclusion.

## Data And Method

Previous researchers considered transportation as the engine of the growth of an economy to investigate the transport-environment nexus (6, 16, 39). Moreover, prior studies considered environmental degradation as a major indicator resulting in health expenditures (21, 22, 46, 47). Thus, this study follows the two streams of literature and bridges them to contribute to the literature. This study collected time-series data from 1990 to 2018 in the United States and China to investigate the associations from transportation to environment to health indicators. The study uses air freight, CO2 emissions, health expenditure as a share of the gross domestic product (GDP) as the proxies to measure transportation, environment, and health. CO2 emissions are measured in carbon dioxide emissions per unit of GDP based on 2010 prices. Air freight is the volume of cargo carried on each flight stage, measured in metric tons times kilometers traveled. Regrading the data source, the air freight and CO2 emissions are retrieved from the World Bank; the health expenditure as a share of the gross domestic product is retrieved from the Organization for Economic Co-operation and Development and the China Health Database for the United States and China, respectively. The time series of air freight has been processed by Logarithm as Logarithmic transformation reduces the effect of the heterogeneous problem where the dynamic range of a variable exists. Table 1 reports the summary statistics of the variables examined in this study. Jarque-Bera statistics indicate that air freight, CO2 emissions, and health expenditure for the two countries are normally distributed.

**Table 1:** Summary Statistics of the variables.

**Country**	**Variables**	**Mean**	**Max**	**Min**	**STD**	**Skewness**	**Kurtosis**	**J-B**
China	TR	8.659	10.136	6.707	1.077	−0.264	1.696	2.391
	ENV	1.605	2.625	0.948	0.441	0.755	2.929	2.764
	HE	1.093	1.870	0.630	0.440	0.652	1.802	3.794
The United States	TR	10.296	10.669	9.580	0.350	−0.872	2.362	4.170
	ENV	0.411	0.538	0.276	0.086	−0.071	1.667	2.171
	HE	14.295	16.844	11.239	1.849	−0.030	1.448	2.914

^*^Represents a significant level of 10%. TR, ENV, and HE refer to air freight, CO2 emissions, and health expenditure, respectively.

This study visits the long-run cointegration and causal relationship between transportation to the environment to health through applying bootstrap ARDL with a Fourier function. The pioneer proposing the ARDL model is (48). Following the knowledge of the ARDL model, (49) proposed the bootstrap ARDL model. Recently, (50) developed the bootstrap ARDL model with the Fourier approximation methodology. The pros of the bootstrap ARDL model with the Fourier function over conventional cointegration tests are as follows. First, the bootstrap ARDL test, different from the conventional ARDL bounded test, allows for (weak) endogeneity of two or more variables of interest, which is valuable for handing feedback from the dependent to the independent variable. Second, building suitable power and size properties, the bootstrap test is more rigorous than the asymptotic test while testing the hypothesis/conditions for cointegration. Third, a lagged independent variable is incorporated into the model such that an additional test is added to support the current F and t cointegration tests suggested by (48). Finally, the Fourier function technique overcomes the drawbacks of the traditional tests for cointegration by allowing structural breaks whose numbers, location, or forms are unknown a priori.

To examine the relationship between transportation, the environment, and health, the following model is used in this study.


(1) $$\begin{array}{l} \text{Δ}y_{t}=c+\gamma_{1}\sin\left(\frac{2\text{π}kt}{T}\right)+\gamma_{2}\cos\left(\frac{2\text{π}kt}{T}\right)+\delta_{1}y_{t-1}+\delta_{2}x_{t-1}\\ \;+\delta_{3}z_{t-1}+\sum_{i=1}^{p-1}\lambda_{1}\text{Δ}y_{t-i}+\sum_{i=1}^{p-1}\lambda_{2}\text{Δ}x_{t-i}+\sum_{i=1}^{p-1}\lambda_{3}\text{Δ}z_{t-i}+e_{t} \end{array}$$

where c is a constant, Δ is the operator for forwarding difference; *p* indicates the lag order; *i* represents the lag index; *t* stands for the periods; *e*_*t*_ is the disturbance term; *x* and *z* are the independent variables; *y* denotes the dependent variable; λ_1_, λ_2_, λ_3_ , δ_1_, δ_2_, δ_3_ are coefficients of the lagged variables, and T represents the sample size.

The setting regarding the Fourier approximation procedure follows the study of (50). For more details on the evolution of the equations, please refer to the supplementary material.

## Results

### The Unit Root Tests

Three common procedures for testing unit root tests, that is, KPSS, ADF, and PP, were conducted to test the variables for stationarity. The null hypothesis for the first and second ones state that a time series has a unit root. If the null hypothesis is rejected, the time series is stationary. Otherwise, it is not stationary. In Table 2, the results indicate that the three variables, air freight, CO2 emissions, and health expenditure are integrated at I (1) order in both China and the United States. A study by (49) suggests that the bootstrap ARDL model is appropriate for testing the co-integration relation between the variables if all the variables are not at I (2) order or above.

**Table 2:** Results of the unit root test.

		**I(0) level**			**I(1) first differences**		
**Country**	**Variables**	**ADF test**	**PP test**	**KPSS test**	**ADF test**	**PP test**	**KPSS test**
China	TR	0.44 (1)	0.42 (1)	0.68 (0)^**^	−5.13 (1)^***^	−5.22 (1)^***^	0.23(0)
	ENV	−1.14 (4)	−2.26 (1)	0.63 (0)^***^	−1.50 (4)	−3.46 (1)^***^	0.26(0)
	HE	0.93 (1)	0.33 (1)	0.55 (0)^**^	−3.17 (4)^**^	−3.14 (1)^**^	0.43(0)*
The United States	TR	−2.07 (1)	−2.26 (1)	0.61 (0)^**^	−4.98 (1)^***^	−4.98 (1)^***^	0.35(0)*
	ENV	0.81 (2)	0.13 (1)	0.68 (0)^**^	−6.86 (1)^***^	−8.11 (1)^***^	0.08(0)
	HE	−0.65 (1)	−1.06 (1)	0.67 (0)^**^	−3.36 (1)^**^	−3.36 (1)^**^	0.10(0)

^***^Represent 1% significance level and ^**^ denote significance in 5%. TR, ENV, and HE refer to air freight, CO2 emissions, and health expenditure, respectively. The value in parentheses indicates the optimal lag length of the ADF and PP tests as selected by the Schwert (2002) criteria or bandwidth determined by the KPSS test.

### Cointegration Test

This study applies the bootstrap ARDL model with a Fourier function test to explore the long-run relationship between transportation, environment, and health in China and the United States. The model was built based on error-correction models without restrictions where the optimal order of the lags for the variables was determined according to the Akaike and Schwarz information criteria used in other empirical studies (48, 51). The test statistics generated in the bootstrapping process determine whether the cointegration between variables exists or not. As shown in Table 3, F_a_ is the calculated F test statics for the coefficients of y_t−1_, x_t−1_, and z_t−1_. Additionally, t_dev denotes the *t*-test statistics for the dependent variable, and F_indev is the F-test statistics for the independent variables. Fa, t_dev, and F_indev statistics are checked to see whether the statistic values exceed their corresponding critical values. The null hypothesis of no-cointegration was rejected since all three tests are statistically significant. In Table 3, we find the values of Fa, t_dev, and F_indev statistics exceed corresponding critical values in both China and the United States when health expenditure is taken as the dependent variable while air freight and CO2 emissions are taken as independent variables.

**Table 3:** Cointegration between variables.

**Country**	**Dv|Inv**	**F_**a**_**	F*	**t_dev**.	t*_dev.	**F_indev**.	F*_indev.	**Conclusions**
China	HE| ENV,TR [3]	5.567**	3.633	−3.735**	−2.246	8.309**	4.154	Cointegration
	ENV| TR, HE [3]	2.940	4.562	−2.669	−2.551	4.023	5.099	No cointegration
The United States	HE| ENV,TR [2]	10.683***	4.069	−4.078***	−1.543	16.02***	3.999	Cointegration
	ENV| TR, HE [3]	4.080	5.830	−0.908	−3.435	3.110	6.439	No cointegration

*The number in bracket represents the optimal lag selected by AIC. TR, ENV, and HE refer to air freight, CO2 emissions, and health expenditure, respectively*.

### Granger Causality Test

Table 4 reports the results of granger causality test. In the case of China, the positive causality between air freight and CO2 emissions runs from the former to the latter, implying that the growth of transportation leads to environmental degradation, which is in line with major empirical findings (5–7). The results also show a positive causality running from CO2 emissions to healthcare expenditure, indicating that environmental degradation causes increased healthcare expenditure, which is in line with major empirical findings (3). Regarding the case in the United States, the negative causality runs from air freight to CO2 emissions, which implies that the development of transportation improves the quality of the environment, which is in line with previous empirical findings (52). We also find the results that CO2 emissions negatively impact healthcare expenditure, indicating that as the quality of the environment is improved, the spending on healthcare increases, which is in line with major empirical findings (21, 22).

**Table 4:** The Results in granger causality in short term.

**Country**	**ΔENV equation: ΔTR F-statics, (p-value) (Sign)**	**ΔHE equation: ΔENV F-statics, (p-value) (Sign)**
China	2.858^*^(0.090) (+)	11.377^***^(0.001) (+)
The United States	5.251^**^(0.019) (-)	5.046^**^(0.022) (-)

^***^, ^**^, and ^*^ represent the levels of significance in 1, 5, and 10%, respectively. TR, ENV, and HE refer to air freight, CO2 emissions, health expenditure, respectively.

## Discussion and Conclusion

The study examines the transportation, environment, and health dynamics in China and the United States for the time period 1990–2018. To do this, we applied the bootstrap ARDL with a Fourier function procedure to test cointegration and confirm the presence of the long-term relationship between transportation, environment, and health. This study enriches current literature by providing new evidence where we apply the relatively rigorous method to confirm the long- and short-term transportation, environment, and health dynamics. Such relationships of cointegration in transportation, environment, and health dynamics provide useful insights for making proper strategies to develop a healthy and productive economy in the long term. Furthermore, the directions of the causal relationships among the variables are identified, which provides useful insights for policymakers to propose better policies to mitigate environmental degradation and accumulate human capital in the process of economic growth.

Our results confirm the existence of transport-induced EKC phenomenon where the effect of transportation on the quality of environments is different depending on the stage of development in an economy. Within the different stages of development, human beings in society and business activities have various consciousness of environment and health. Our empirical findings provide important implications for policymakers. First, in the context of China, the positive causality between transportation and CO2 emissions indicates that augmenting the development of transportation increases environmental degradation. The findings imply that the process of economic growth is responsible for environmental pollution in China as transportation is the engine of economic growth. Residents pursue an improved quality of life that is suggestively dependent on efficient transportation as a result of the improvement in their economic circumstances. Apart from that, the logistic system, which plays a critical role in the expansion of the economic system, needs an effective transportation system, which raises the demand for passenger and freight transportation services. Thus, one of the causes of environmental degradation is the growth of vehicular traffic flow in urban areas and congestion commonly happens in most major cities in China. The massive growth in the number of vehicles on the road has led to a surge in oil consumption and ejection of harmful gases such as carbon dioxide. On the other hand, the manufacturing and industrial sectors contribute major output to Chinese economics. Specifically, the logistic industry contributing to important added value in transferring products in supply chains has witnessed significant expansion since the logistic industry is a supporting, crucial, and leading industry of economic development. Thus, the increased demand for passenger and freight transportation services both leads to increase in transport-related infrastructure and activities and generates more CO2 emissions in the environment. Another finding for China is that environmental degradation leads to boosting the demand for care of health. Specifically, air pollution has a negative influence on health as the poor environment quality causes illnesses. Previous studies (53, 54) indicated that air pollution is one of the main causes that increase the mortality and morbidity of cardiovascular disease. Thus, the worse the quality of the environment, the more tangible and intangible costs that residents should pay, including healthcare expenditure and health outcomes.

Second, in the context of the United States, the negative causality between transportation and CO2 emissions. The findings imply that the growth of the economy comes up with a better environment in the society in the United States. Specifically, economic growth increases the demand for freight and passenger service in transport since residents with higher income pursue a better quality of life. In recent decades, citizens have grown more aware and concerned about air pollution. This has led to an increasing consciousness that we can no longer disregard the environment in the process of economic development. (55) indicated that the problem of air pollution has been a concern by the broader communities in urban areas such as Pittsburgh. The government in the United States put effort into advanced renewable energy that is more eco-friendly on the environment rather than traditional energy generation. International Energy Agency (56) reported that renewable energy accounts for 12% of transport fuel demand growth from 2018 to 2023 in the world. Specifically, the United States produce biofuel energy of about 65 billion in 2020 liters, which is the major part of the sum worldwide (International Energy Agency, 2021). In the transport sector energy demand, biofuels still hold an almost 90% share of total renewable energy in 2023 (56). The effort in the development and use of advanced renewable energy rather than traditional energy generation supports a better quality of the environment in the United States. Another finding for the United States is that improved quality of environment leads to increased healthcare expenditure. The results align with previous research (21, 22). The EKC links economic growth to the environment, which has been identified as a primary factor justifying the evidence that improved quality of environment leads to more spending on healthcare. For the United States as a developed country, the residents with relatively high per capita income and quality of life could demand better nutrition, medical care, and medicines. Additionally, the residents could increase preventive health expenditures to strengthen their health itself since health expenditures include preventive and curative health services. Kamal and Hudman (57) reported that preventive care spending is higher in the United States than in many comparably wealthy countries.

We recommend that the Chinese government should enhance its transportation system and encourage the auto industry to introduce more hybrid-type cars. With a better transportation system, the residents could rely less on private vehicles for moving, and thus, congestion could decrease. The improved environmental quality in a region not only benefits the humans who live there but also promotes the development of the region where skilled workforces preferring better quality of life commonly move. The United States government should continue to put effort into investment in advanced renewable energy and support the use of hybrid or energy-efficient automobiles. The government is suggested to deliver the residents and enterprises the knowledge regarding a variety of initiatives, such as disaster preparedness, nutrition and education programs, disease prevention programs, and epidemiological surveillance to promote population health and wellbeing. This study takes the environment as the factor impacting health; however, other factors are considered to validate related theory where health could be influenced by other factors. In the future, it is suggested to investigate more issues such as energy consumption that would influence the environment. Moreover, a more deep understanding of the relationship between healthcare expenditure and health status would be investigated.

## Data Availability Statement

Publicly available datasets were analyzed in this study. This data can be found here: https://data.worldbank.org/.

## Author Contributions

C-FW and M-CL contributed to the research topic, research model, and the statistical analysis and writing. All authors contributed to the article and approved the submitted version.

## Funding

This study were supported by Hubei University of Economics with the grant number of XJ201901, XJ201902, 11024225, and Research Center of Hubei Logistics Development.

## Conflict of Interest

The authors declare that the research was conducted in the absence of any commercial or financial relationships that could be construed as a potential conflict of interest.

## Publisher's Note

All claims expressed in this article are solely those of the authors and do not necessarily represent those of their affiliated organizations, or those of the publisher, the editors and the reviewers. Any product that may be evaluated in this article, or claim that may be made by its manufacturer, is not guaranteed or endorsed by the publisher.
